# Auricular vagus nerve stimulator for closed-loop biofeedback-based operation

**DOI:** 10.1007/s10470-022-02037-8

**Published:** 2022-05-10

**Authors:** Babak Dabiri, Klaus Zeiner, Arnaud Nativel, Eugenijus Kaniusas

**Affiliations:** Institute of Electrodynamics, Microwave and Circuit Engineering, Vienna University of Technology, Vienna, Austria

**Keywords:** Auricular vagus nerve, Optimization, Closed-loop control, Hardware, Vagus nerve stimulation

## Abstract

Auricular vagus nerve stimulation (aVNS) is a novel neuromodulatory therapy used for treatment of various chronic systemic disorders. Currently, aVNS is non-individualized, disregarding the physiological state of the patient and therefore making it difficult to reach optimum therapeutic outcomes. A closed-loop aVNS system is required to avoid over-stimulation and under-stimulation of patients, leading to personalized and thus improved therapy. This can be achieved by continuous monitoring of individual physiological parameters that serve as a basis for the selection of optimal aVNS settings. In this work we developed a novel aVNS hardware for closed-loop application, which utilizes cardiorespiratory sensing using embedded sensors (and/or external sensors), processes and analyzes the acquired data in real-time, and directly governs settings of aVNS. We show in-lab that aVNS stimulation can be arbitrarily synchronized with respiratory and cardiac phases (as derived from respiration belt, electrocardiography and/or photo plethysmography) while mimicking baroreceptor-related afferent input along the vagus nerve projecting into the brain. Our designed system identified > 90% of all respiratory and cardiac cycles and activated stimulation at the target point with a precision of ± 100 ms despite the intrinsic respiratory and heart rate variability reducing the predictability. The developed system offers a solid basis for future clinical research into closed-loop aVNS in favour of personalized therapy.

## Introduction

**Fig. 1:**
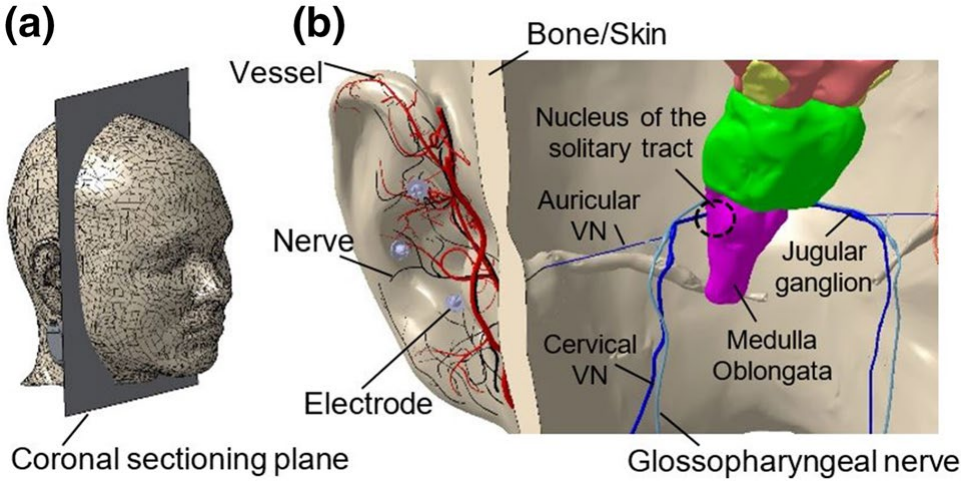
**a** Location of the coronal sectioning plane of the head to show the relevant VN network in **b**. The posterior side of the sectioned head with three needle electrodes in the cymba conchae region of the external ear, which stimulate aVN endings. The stimulation is projected by the aVN branch extension from the external ear to the vagus nerve (through external auditory meatus) into the jugular ganglion and eventually to the nucleus of the solitary tract. Vessels and nerves are shown in red and black, respectively. External ear model with the associated vessels and nerves are taken from [14, 15], the head model from [16]

The vagus nerve, with about 80% afferent fibers, is part of the autonomic nervous system and thus is heavily involved in sensory and regulatory mechanisms of the body-brain interaction [1]. One of the easily accessible peripheral point to the afferent vagus nerve is the cymba conchae of the external ear [2], with reported 100% innervation by the auricular branch of the vagus nerve (aVN) [3]. Therefore, the peripheral stimulation of this areas can be expected to connect directly to the brainstem. From an anatomical point of view, aVN endings in the pinna join the vagus nerve at the jugular ganglion level (just outside the cranium) through the external auditory meatus, see (Fig. 1) [4, 5]. Frangos et al. proved activation of the main visceral sensory nucleus of the solitary tract in the medulla oblongata as a result of the electrical stimulation of the cymba conchae [6].

Auricular vagus nerve stimulation (aVNS) is a non- or minimally-invasive therapeutic solution to treat epilepsy [7] by inducing stabilized neuroplastic modifications of brain network [8], chronic low back pain [9], psychiatric disorders [10], and many more systemic disorders [11]. From a physiological perspective, the effects of aVNS are hypothesized to induce systemic changes of the sympathovagal balance of the autonomous nervous system [4, 13], resolution of maladaptive and acceleration of neuroplasticity, antinociceptive and anti-inflammatory effects [4]. Recently, aVNS relevance was hypothesized to ameliorate Covid-19 related acute respiratory distress syndrome [12].

While transcutaneous aVNS uses non-invasive surface electrodes in the ear with a diffuse stimulation field [2], the percutaneous aVNS uses miniature needle electrodes placed near aVN or its endings [17] with a more targeted stimulation field [18]. The minimally invasive percutaneous aVNS requires also less stimulation energy than the transcutaneous aVNS since the epidermal skin does not act as a current barrier; in addition, the percutaneous aVNS provides maximum efficiency for the applied stimulation pattern [19, 24]. Unlike the cervical vagus nerve stimulation with invasive electrodes and potentially severe side effects (e.g., chronotropic effects [20]), the percutaneous aVNS is associated with mild side effects only (e.g., local skin irritation) [21].

The stimulation parameters are usually selected in an empirical way, based on perception to attain a subjectively comfortable intensity (e.g., tingling) [22, 23]. However, this method of stimulation parameter adjustment depends highly on the used stimulation patterns, may yield over-stimulation and/or under-stimulation as well as suboptimal temporal interference with inner physiological rhythms, and thus suboptimal therapeutic outcomes [4, 24]. For instance, sympathetic or parasympathetic response to aVNS seems to be frequency and pulse width dependent. Namely, the relatively high frequencies (20–250 Hz) and short pulses tend to excite the relatively thick myelinated Aβ fibers and thus to activate the parasympathetic system. The relatively low frequencies (0.5–10 Hz) and elongated pulses tend to activate both the relatively thick and thin fibers, and thus activate also the sympathetic system [4, 25].

The complex and multifactorial body regulatory system challenges the application of aVNS. For instance, continuous or intermittent aVNS, with mild to strong intensity, might provoke compensatory sympathetic system in response to aVNS induced bradycardia [26] and thus a sympathovagal misbalance. Therefore, in order to account for the physiological state of patients, aVNS requires a physiological feedback to optimally adjust the stimulation settings, i.e., not only its magnitude but also its timing with respect to cardiorespiratory body rhythms. Our recently published work [2] shows that different stimulation patterns act differently on the human body.

Recent works dealing with the closed-loop vagus nerve stimulation have already shown that it is possible to adaptively control the heart rate in sheep or pigs [27, 28], as well as to reduce seizure frequency in epileptic patients with heart rate triggered stimulation bursts [29]. However, these studies all used invasive cervical vagus nerve stimulators. Non-invasive closed-loop systems are still sparse at this time and use different signals as input; e.g., in a study by Cook et al., transcutaneous vagus nerve stimulation was triggered by muscle activation to enhance motor recovery in neurorehabilitation [30].

Hence, a biofeedback-based stimulation hardware is required for aVNS which can individualize stimulation parameters based on real-time physiological data. In this work, we present a prototype hardware setup (experimental and standalone) which can not only integrate internal (and external) sensors but can also process data in internal (and external) units in real-time, offering a solid basis for a complete closed-loop control of aVNS. This enhances the functionality of the currently available aVNS systems, which either stimulate with fixed strength or offer a subjective adjustment of the stimulation strength; however, the timing is not adjusted at all.

This article extends the authors’ previous work [31], which shares the proposal of a framework for a multifunctional auricular vagus stimulator for closed-loop application. It extends state-of-the-art while considering available aVNS modalities and presents more details on the aVNS stimulation pathway projecting into the brain using an in-silico model (section I). Further, it amends respiration-based measurements (summarized in Fig. 7) and presents a standalone setup using embedded biosensors for cardiac and respiration cycles (section III).

## Methods

**Fig. 2:**
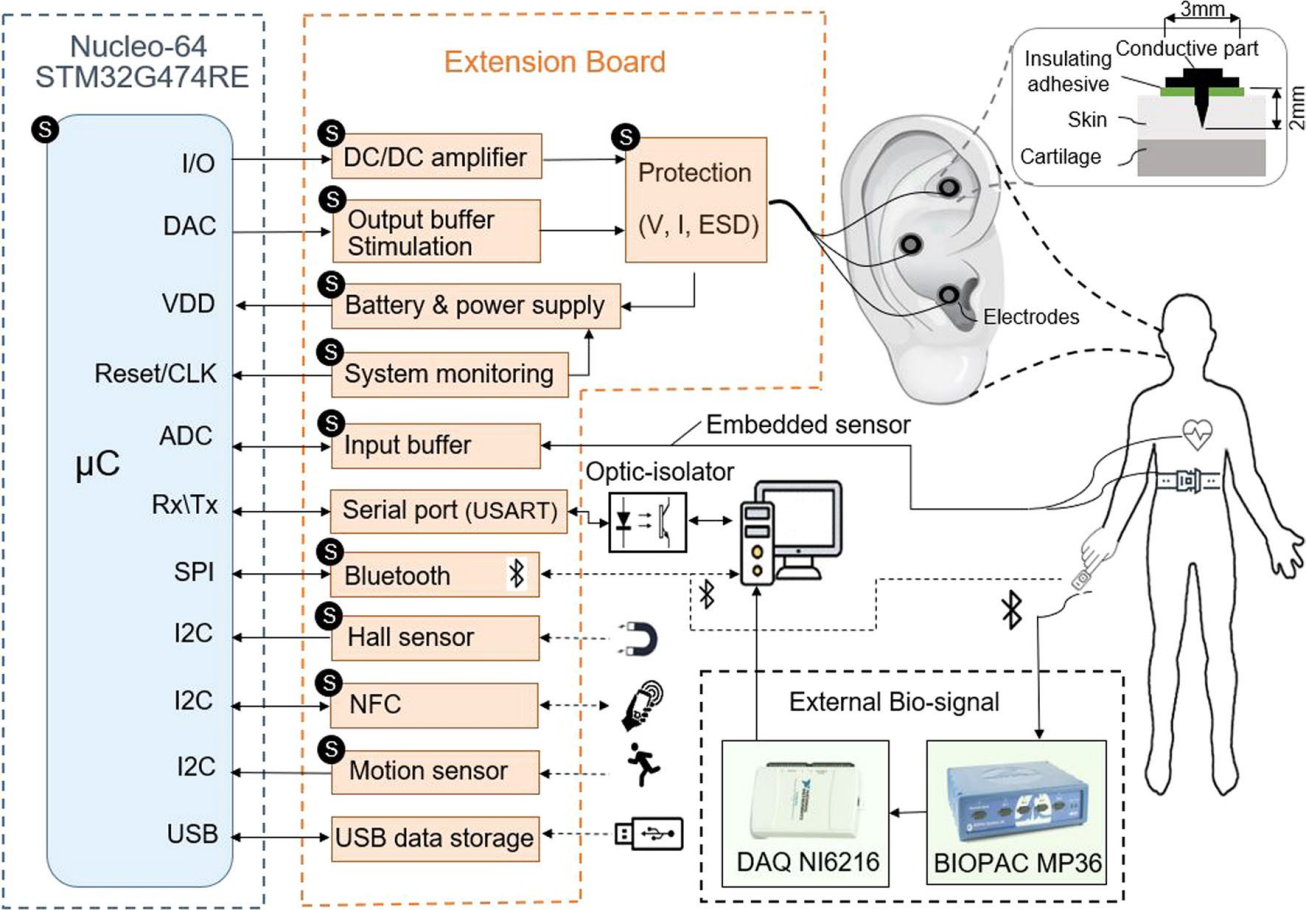
Block diagram of the multifunctional auricular vagus nerve stimulator for experimental and standalone setup: microcontroller (blue), designed extension hardware (orange), external data acquisition module (green), the human auricle with inserted stimulation electrodes, and electrode inserted into the ear (top right). The blocks marked with “S” are used for standalone setup

**Fig.3:**
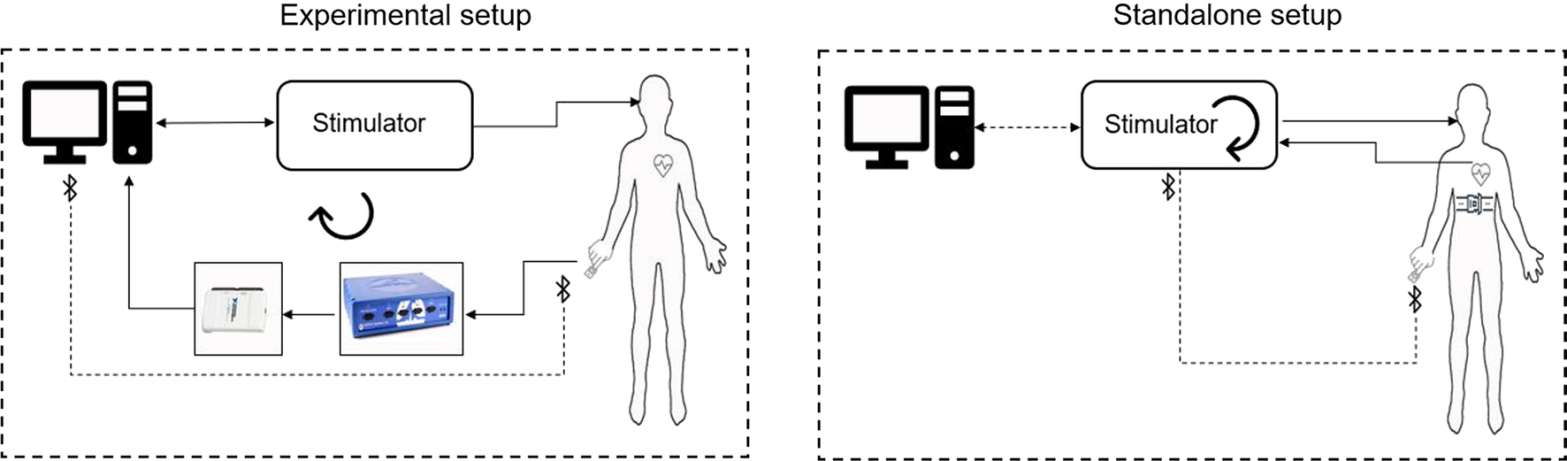
The closed-loop aVNS with experimental and standalone setups

We designed a system which can be operated in two modes: (1) experimental setup with external biosignal acquisition and a PC running a MATLAB GUI application (R2020b, the MathWorks Inc., Natick, MA, USA), and (2) standalone setup with a microcontroller unit (MCU) as the main processor, with integrated or Bluetooth-connected biosensors for portable closed-loop wearable aVNS (Fig. 2). Basically, the experimental setup (1) can be used for verification and implementation of the whole procedure for the standalone application in MCU (2). The closed-loop stimulation is shown in (Fig. 3) for both setups. In the experimental setup, feedback biosignals are acquired from body and processed first in PC. Then stimulation is initiated via aVNS stimulator. In contrast, in the standalone setup, the biosignal is directly acquired and processed within the stimulator while the effective stimulation can be initiated either with or without mediation via PC.

### Processor

As a base of the stimulator a development board STM32 Nucleo-64 with STM32G474RE MCU was employed, extended with a custom peripheral board. The series STM32G474RE features an Arm Cortex-M4 core with DSP and FPU, 170 MHz clock rate with 512Kbytes of Flash memory, Math Accelerator, 17 timers, and numerous communication interfaces (SPI, I2C, USART, USB, etc.). Especially, seven 12-bit DAC channels and seven ultra-fast rail-to-rail analog comparators were needed to drive three independent stimulation channels subjected to necessary safety measures while minimizing number of external DAC and analogue components.

### Stimulation parameters

**Fig. 4:**
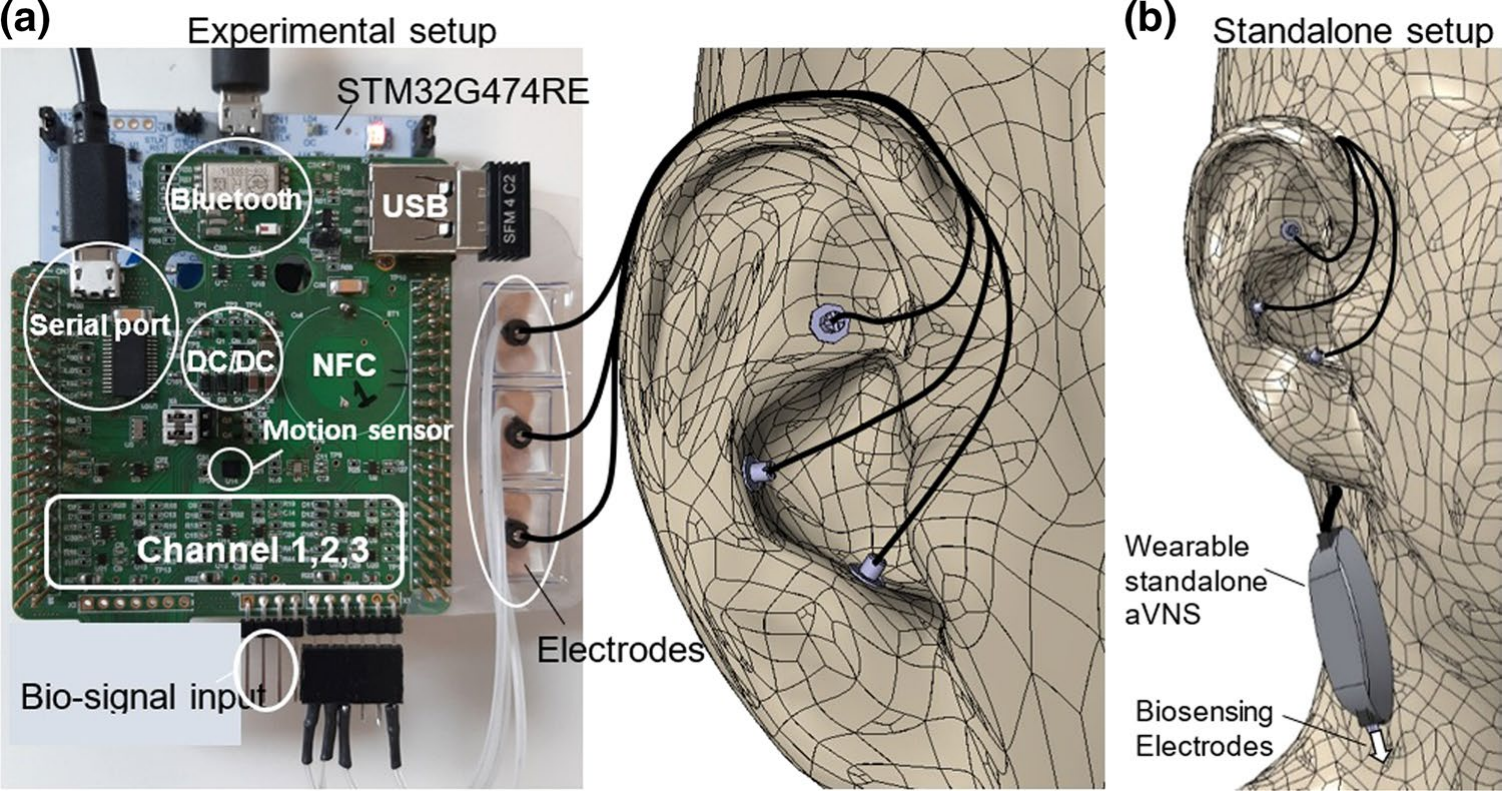
**a** Experimental setup: extension board (green) is attached to the Nucleo-64 development board (white) in the form of a sandwich panel while three output channels stimulate the cymba conchae region of the external ear using miniature needle electrodes. **b** Standalone setup for wearable applications

The STM32 Nucleo board communicates with its peripherals to control aVNS parameters like the stimulation pattern (mono-phasic, bi-phasic, or tri-phasic [24]), current/voltage amplitude, number, or width of the pulses within bursts, and the duty cycle (channels 1–3 in Fig. 4a). In the experimental setup, the stimulation pattern is defined in MATLAB and kept constant throughout the stimulation (here, tri-phasic) and sent to the microcontroller via (optically isolated) serial port or Bluetooth. Within the hardware, the stimulation amplitudes are adjusted in two subsequent stages: first, in the range of 0–1.2 V in the DAC channels output (with 12 bits resolution and fully differential output voltage) and, second, by a Switch Boost DC\DC converter (TPS61040DRVR, Texas Instruments) in the range of 3.3–6 V. The current passing through the patient is sensed with the current to voltage converters while the current’s maximum is limited to 1 mA to provide overcurrent protection in terms of patient safety. Likewise, the applied voltage is sensed by ADC to compare with the target values to allow continuous surveillance in terms of patient safety.

### Protection and safety

Protection is considered from the patient and device side, in line with the medical device basic safety and essential performance standard EN 60,601–1. The optically isolated serial port or wireless Bluetooth interface avoids possible conductive hazardous current leaks from PC and other powered peripherals connected to the patient. The applied voltage and current to the patient are constantly monitored, and—in case of exceeding boundary values—the stimulation power supply is shut down immediately (without microcontroller contribution) to protect the patient. Moreover, the potential electrostatic discharge from the body to the device (e.g., due to defibrillation) is prevented by varistors and Zener diodes, avoiding damage to circuitry components. The system monitoring takes care of software failures by stopping the stimulation and issuing warnings, in line with the medical device software standard EN 62,304, see (Fig. 2).

### Usability

A well-developed design should consider ease of use for both patient and physician, in line with the usability engineering standard EN 62,366–1. Hence, an integrated Hall sensor, as well as near field connection coil with NFC EEPROM, represent possibilities for the patient and physician to exchange data with the stimulator. Not only the stimulator but also the biosignal acquisition can be controlled on demand while offering contactless access to stimulation and biosignal data.

### Connectivity and portability

(Figure 4) shows all subunits implemented in the experimental set-up. The Bluetooth module (BLUENRG-M2A, STMicroelectronics) is used with the two aims, to attain portability of the device during experiments and wireless connection to PC with conductive isolation. Furthermore, it can be used for communication with external wireless bio-sensors for portable applications. The serial port connection provides an independent direct communication between microcontroller and PC, in addition to that from the integrated serial port in STM32 Nucleo board, to interact with external software like MATLAB in real-time, especially when using the debug mode, as shown in the block diagram of (Fig. 2). Moreover, dimensions of the experimental setup 7 × 7 × 1 cm^3^ (l × w × h) and of the standalone setup 4 × 3 × 1 cm^3^ with Li-Ion battery (3.7 V/1000mAh for experimental setup and 3.7 V/210mAh for standalone)—incl. temperature, over current, and voltage protection—favor their portability, wearability, and long-term application (Fig. 4a, b). However, for the minimum standalone system, NFC, motion sensor, and Hall sensor can be omitted.

### Sensing

A motion sensor (ISM330DHCXTR, STMicroelectronics) provides positioning information of the stimulator and the patient to monitor and account for patient movements, with embedded proper filtration strategies of monitored biosignals.

The proposed hardware setup can acquire biosignals (e.g., electrocardiography (ECG), pulse plethysmography (PPG) and/or respiration) directly with the integrated hardware and/or with external biosensors (e.g., BITalino, PLUX Wireless Biosignals S.A) via Bluetooth modules and/or external monitors (e.g., BIOPAC, BIOPAC Systems, Inc., CA, U.S.), and even send data to PC via ADC (DAQ NI6216, National Instruments, Austin, Texas,U.S.) for signal processing in real-time. These biosignals act then as biofeedback in the control loop. The type of utilized biosignal is determined by the application requirements, e.g., PPG can be used for patients with peripheral blood perfusion disorders, whereas ECG and respiration for patients with cardiac or baroreflex disorders. For instance, aVNS was applied for the respiratory-gated aVNS [23].

Internal sensing may include hardware components for ECG (e.g., MAX30001, Maxim Integrated), PPG (e.g., MAX86150, Maxim Integrated), and respiration monitoring (e.g., realized as a sandwich panel on the development board). External signal acquisition is also recommended for experimental applications to visualize biosignals in the laboratory setup. Internal sensing is more appropriate for long-term studies to minimize patient’s restrictions and increase his/her comfort. The standalone set-up can be attached to the patient, for instance, as an upper-armband for the duration of several days.

### GUI application

**Fig. 5:**
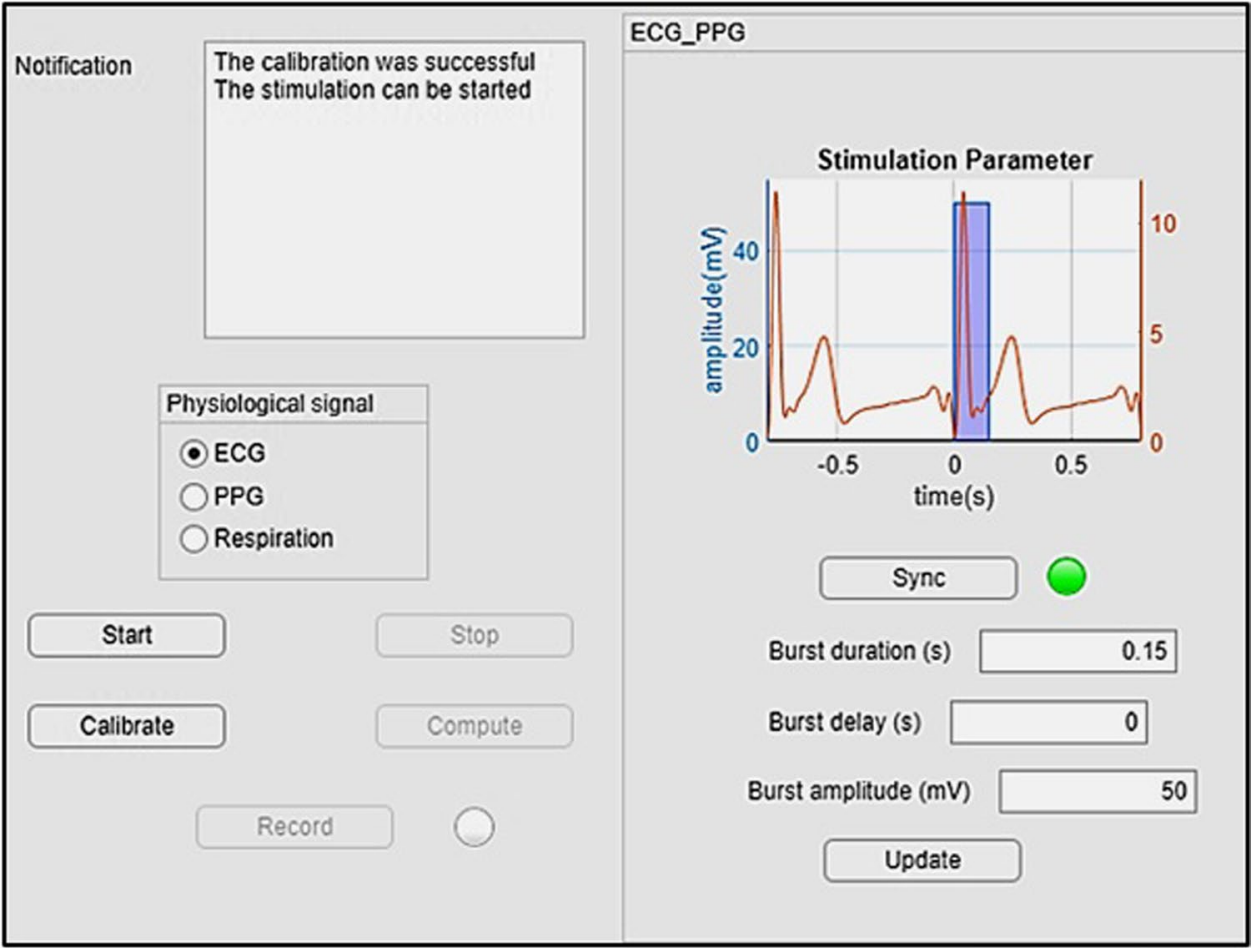
Designed GUI application with MATLAB

For the targeted stimulation in a laboratory setting, a GUI application was designed in MATLAB (Fig. 5). For calibration and avoidance of motion artifacts, the application first acquires a selected biosignal (ECG, PPG or respiration) without any motion artefacts for 20 s during normal and deep breathing cycles. Then, the biosignal is split into individual cardiac or breathing cycles in order to create a single template per single cardiac or breathing cycle. The template contains the characteristics of the biosignal form, namely, the slope variability for R-peak in ECG, for the systolic phase in PPG, or the inspiratory phase in normal and deep breathing.

Once the calibration is done, the stimulation process can be started. In this process, every 50 ms a frame of 25 data points for ECG and PPG, and every 200 ms a frame of 100 data points for respiration is acquired and filtered with a FIR low pass filter (Barlett–Hanning-Windows) at a cutoff frequency *f*_C_ = 15 Hz to eliminate possible noise. Then the slope of each filtered segment of the biosignal is compared to that in the templates derived in the calibration. If the actual slope is within the interquartile range 25–75% of (a) all R-peaks used for template creation in ECG, (b) all systolic phases used for template creation in PPG, or (c) all inspiration phases used for template creating in respiration, then the actual data segment is considered being valid, i.e., without motion artefacts. In addition, the fulfillment of this condition indicates the time point within the cardiac cycle in ECG, PPG, or respiration phase in the respiration signal.

In order to initiate aVNS at a specific target point of the cardiac cycle (e.g., Q-peak with a specific time delay), a detected reference point (e.g., R-peak in ECG or systolic peak in PPG) of the previous cardiac cycle is required. Then, the stimulation is predicted for the next cardiac cycle and delayed by one cardiac cycle in the real-time application. This procedure considers spontaneous and instantaneous heart rate changes to a certain extent, as opposed to a currently fixed non-individualized stimulation frequency.

There is a systemic delay due to the (software) negotiation with other peripheries, data processing, and filtration procedures, which is computed and explicitly considered for the proper operation of aVNS, i.e., setting the temporal stimulation target. However, the finite heart rate variability and possible unexpected hardware delays prevent a precise reach of the planned stimulation point in time. Therefore, the actual stimulation point is usually distributed around the target point, as illustrated in Fig. 7. aVNS stimulation is activated at the targeted time point for a certain burst duration and then deactivated until the next reference point is detected (i.e., within the next cardiac or respiration cycle). Data is stored for further processing, specifically to tune stimulation parameters based on the body response while stimulating and registering biosignals simultaneously. Moreover, stimulation progress and the rate of successful stimulation activation can be visualized and assessed on request to verify the aVNS procedure in the course of the experiment or at the end of each experiment (with the compute button, see Fig. 5).

## Results and discussion

aVNS prototype was developed and tested for closed-loop feedback applications with biosignals registered using internal and external sensors.

**Fig. 6:**
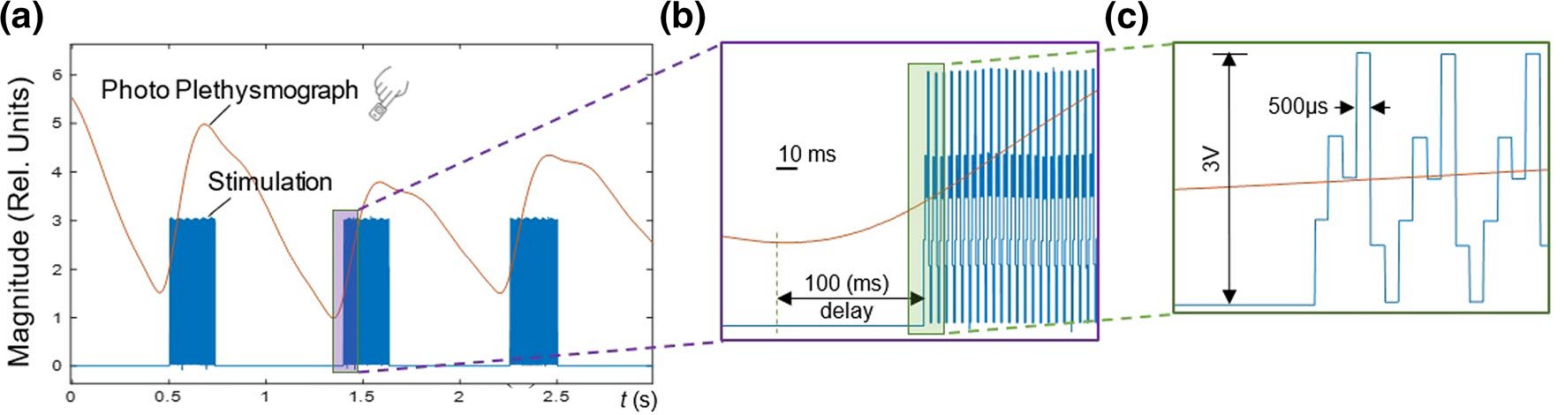
aVNS synchronized with a cardiac biosignal, as **a** based on PPG from the finger. **b** The stimulation started with a delay of 100 ms with respect to the onset of the rising slope of PPG. **c** The applied tri-phasic stimulation pattern (applied to all three electrodes)

**Fig. 7:**
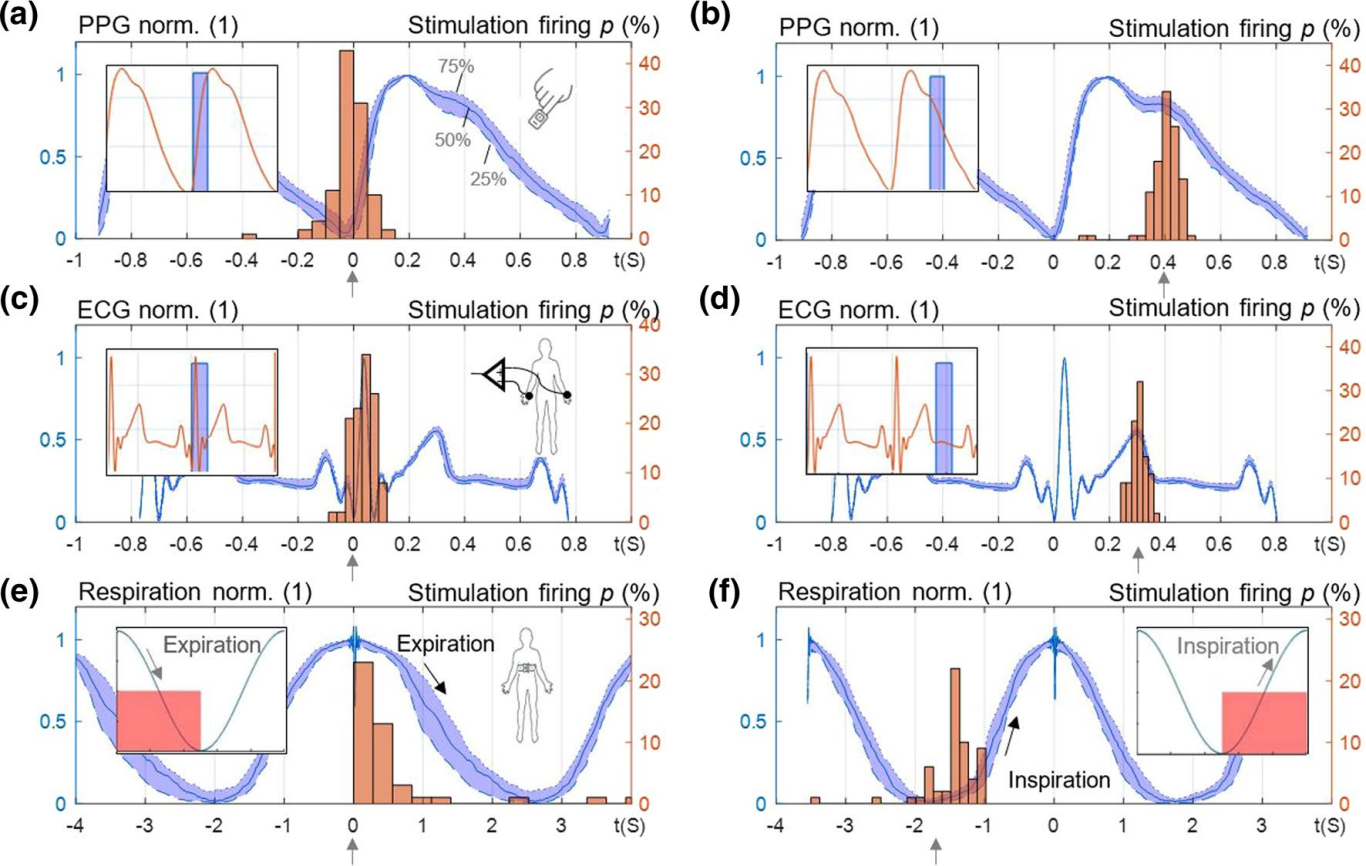
Auricular stimulation synchronized with three different biosignals, i.e., PPG, ECG and respiration. **a**, **b** The distribution of PPG from the finger over the recording duration (interquartile range, in blue) and the density distribution of the stimulation on-sets (in red) with **a** zero delay and **b** 0.4 s delay with respect to the end of the diastolic valley (insets show stimulation bursts along PPG during calibration). The target time instant is marked with arrows. **c**, **d** The distribution of ECG and the density distribution of the stimulation on-sets with **c** zero delay and **d** 0.3 s delay with respect to Q-Peak of ECG. **e**, **f** The distribution of respiration signal and the density distribution of the stimulation on-sets with respect to **e** start of expiration phase and **f** start of inspiration phase. The results are given for the experimental setup

As shown in (Fig. 6), a PPG signal from the finger—as acquired by BIOPAC and sent to the PC—is used as biofeedback for the proposed closed-loop aVNS. In (Fig. 6a–c) aVNS stimulation starts with 100 ms delay with respect to the onset of the ascending slope of PPG, the local systolic phase, as detected by MATLAB in the real-time loop. An adjustable arbitrary delay (e.g., within the cardiac cycle range) with respect to the specific cardiac reference point allows a targeted physiological point-based stimulation (e.g., within systolic or diastolic phases) to investigate stimulation effects in more detail.

(Figure 7) shows the designed system processing with real-time targeted stimulation based on cardiac events from PPG (Fig. 7a, b), from ECG (Fig. 7c, d) as well as from respiration belt (Fig. 7c, d). While ECG as feedback biosignal reflects the electrical cardiac activity at the level of the heart, PPG reflects the local peripheral mechanical cardiac activity. (Figure 7) shows the distribution of the recorded ECG, PPG and respiration signals over the whole measurement duration of 3 min (with the indicated interquartile range from 25 to 75%, in blue) as well as the distribution of the resulting time instants of the stimulation on-sets (histogram bars, in red) around the target time instant (= 0 s and 0.4 s in Fig. 7a, b for PPG, 0 s and 0.3 s in Fig. 7c, d for ECG, expiration and inspiration phase in Fig. 7e, f). Here the stimulation magnitude (voltage) and burst duration (number of pulses) are kept constant at 50 mV and 0.15 s, respectively, to demonstrate only the synchronization between aVNS and cardiac intervals (Fig. 7a–d insets), and respiration phases (Fig. 7e, f insets). As expected, the mean starting point of the resulting stimulation is located at the targeted time instant. However, due to the finite heart rate variability, the actual stimulation starts within ± 100 ms standard deviation around this point, which is comparable with the natural variation in the heart period. An advanced predictive algorithm could decrease this standard deviation and make the distribution around the target point even more narrow. For the respiration synchronization, the standard deviation is about ± 400 ms with respect to the onset of inspiration or expiration, which is due to a slower acquisition of respiration data (by factor 4) as compared to ECG or PPG.

The developed experimental setup provides a basis for the closed loop aVNS using internal and external biosensors with concurrent optimization of stimulation parameters based on real time biosignal acquisition. In contrast, the standalone setup uses internal biosensors such as embedded ECG and bioimpedance sensors (e.g., MAX30001, analog front-end solution) with integrated hardware signal processing, providing a portable and more straightforward data acquisition. Here, for instance, both heart and breathing rates are acquired with internal multiplexing between ECG and bioimpedance using the same set of electrodes attached to the patient’s ear. Since the energy consumption for continuous assessment of cardiorespiratory rhythms is crucial in portable applications, ECG and bioimpedance with the consumption of 0.45mW are more favorable than PPG with 1.35 mW (e.g., MAX86150). In addition, most of the biosignal processing is done by embedded hardware which significantly reduces the real time delay, computational load for microcontroller, and allows faster system responses in the standalone than experimental setup. An optimized sampling frequency of *f*_S_ = 20 Hz minimizes the processing and communication times without losing the targeted time-related resolution of cardiac and respiratory signals. Processing and communication delays (in the range of 100 ms) comprise only a fraction of the cardiac cycle and can be compensated internally. Therefore, the control loops stay stable in real-time operation.

The feedback biosignal can be also stored in PC for further processing using MATLAB. The stored data can be analyzed in time and frequency domains to assess the effects of aVNS. For instance, based on the assessed heart rate variability, the stimulation parameters like current amplitude or the number of pulses within bursts can be tuned in MATLAB and sent back to aVNS stimulator, thus personalizing the stimulation and optimizing aVNS therapy. Nonetheless, this therapy is only applied to stable patients and not patients in critical situations.

We have shown that aVNS stimulation can be instantaneously synchronized to specific intervals of diverse human body rhythms, where, for instance, the parasympathetic activity is elevated, such as the expiration phase of respiration, and/or relaxed position [11, 23]. Thus, aVNS can yield synergic and constructive effects on the inner parasympathetic tone of the human body with its regenerative effects. It can be expected that aVNS stimulation throughout the expiration phase of respiration or QRS-complex of ECG may elevate the vagal tone in addition to the vagal baroreceptor input, thus improving therapeutic aVNS efficiency.

## Conclusion

We established a biofeedback-based aVNS system which provides a flexible control of stimulation parameters using cardiorespiratory biosignals of the human body. To the knowledge of authors, none of the existing aVNS set-ups provide such diversity in communication with different biosensors nor consider body rhythms to adapt the stimulation parameters in time and strength. Our hardware can communicate with different types of sensors and external units to personalize aVNS therapy. In future, an appropriate controller for the closed-loop operation is required to optimize stimulation parameters (magnitude, burst duration) with respect to biofeedback signals and therein derived parameters. Further investigations are needed in conjunction with physiological experiments.

A targeted stimulation considering different physiological rhythms in their phase and magnitude could open new venues for detailed investigations of aVNS, such as baroreflex-related aVNS effects. Flexible connectivity with periphery in a compact system increases the precision of the feedback and offers a comfortable and novel research setup for aVNS.

## Data Availability

The raw data supporting the conclusions of this article will be made available by the authors, without undue reservation, to any qualified researcher on reasonable request.
